# Dual mediating effects of changes in daily life and anxiety on the relationship between occupation and depression in Korea during the COVID-19 pandemic

**DOI:** 10.1186/s12889-022-13932-0

**Published:** 2022-08-05

**Authors:** Soo-bi Lee, Ye-bin Jeon, Myeong-Sook Yoon

**Affiliations:** 1 Department of Social Welfare (BK21 FOUR), Jeonbuk National University, Jeonju, South Korea; 2 Department of Social Welfare, Jeonbuk National University, 567, Baekje-daero, Deokjin-gu, Jeonju, Jeollabuk-do 54896 South Korea

**Keywords:** Occupation, Depression, Degree of changes in daily life, Anxiety, COVID-19

## Abstract

**Background:**

A substantial proportion of the world’s population experienced social, economic, and mental health challenges, including considerable changes in everyday life, due to the COVID-19 pandemic. However, these challenges varied in intensity depending upon occupation type and working environment. In this context, this study helps shed light on the effects of occupation type on depression through the mediation of changes in daily life and anxiety as perceived by individual workers through their experiences of the COVID-19 pandemic.

**Methods:**

In total, 68,207 adults (aged 19–65 years) working in the “office” or “service” sectors were analyzed based on the raw data extracted from the 2020 National Community Health Survey conducted by the Korea Disease Control and Prevention Agency. Data analysis was performed using PROCESS Macro (Model 6) for SPSS 25 to examine how depression is affected by occupation type through a dual mediation of the changes in daily life and anxiety caused by COVID-19 as perceived by individual workers during the pandemic.

**Results:**

First, service workers perceived COVID-19-related changes in daily life more acutely than the office workers. Second, service workers felt more COVID-19-related anxiety than office workers, whereby the higher the level of COVID-19-related changes in daily life perceived by the workers, the higher the level of their COVID-19-related anxiety. Finally, service workers’ perceived COVID-19-related changes in daily life more acutely than office workers, which had a positive effect on the level of COVID-19-related anxiety, ultimately increasing depression.

**Conclusions:**

It was found that the impact of a special disaster situation, such as the COVID-19 pandemic, on the perceived changes in daily life and anxiety varies depending on occupation type, which suggests that different occupations have different effects on mental health outcomes. This highlights the need to develop various customized services and policies to promote mental health according to the type of occupation, considering the working environment and work characteristics of those vulnerable to COVID-19 infection.

## Background

Over the past 2 years, the COVID-19 pandemic has exposed a considerable proportion of the population to various changes in daily life and social, economic, and mental health challenges. Under the implementation of intense infection control measures, self-employed businesses have been subjected to restrictions such as limited business hours. Restricted number of people in private gatherings and the shift of schools and private academies from offline to online classes as the main instruction delivery mode were noted during the pandemic. Many people faced a disruption of daily life routines. Furthermore, COVID-19, coupled with the fear of infection and various socioeconomic factors, impacted many spheres of life, such as unemployment, loss of income, differences in academic achievement, interpersonal relationships, and restrictions on freedom [1, 2].

According to the survey results released by the Korean Society for Traumatic Stress Studies [3], the most common cause of stress due to the COVID-19 pandemic is the inability to implement plans. This is followed by financial difficulties due to reduced income or debt, physical and mental health problems, and difficulties associated with academic or job performance due to changes such as telecommuting. This implies that people are experiencing changes across all aspects of daily life due to the pandemic. Owing to these changes in daily life, depression and anxiety levels have been markedly increasing worldwide after the COVID-19 outbreak [3, 4]. In particular, depression is considered the most typical negative mental health outcome caused by the pandemic, signaled by the neologism “corona blues” [5–7]. Depression is a representative indicator of mental health and is a risk factor closely associated with alcoholism, anxiety disorders, and suicide [8, 9].

This situation is supported by some studies that have reported that COVID-19-related changes in daily life affect depression and anxiety [2, 10, 11], especially mental states of those affected by confirmed cases of infectious disease, such as fear of stigma of infection and social rejection, worries about caregiving family, self-reproach for spreading infection, loneliness due to social isolation, pain, and helplessness [12]. Conversely, Cho [13] noted that each person has different perceptions of disaster situations and experiences fear differently according to the degree of acceptance. This suggests that while objective measurement of COVID-19-related changes in daily life is important, it is also worth considering individually perceived changes in daily life—subjective feelings about the changes—as an important predictor of mental health.

During the COVID-19 pandemic, while everyone experiences anxiety and depression along with changes in daily life caused by COVID-19, the level of perceiving these experiences varies to a certain extent depending upon occupation type and working environment [14–18]. Measures to counter infectious diseases inevitably restrict daily and socioeconomic activities. Such restrictions on utilization of public facilities as well as travel and mobility raise concerns about unemployment and dwindling income among workers in the sales/service sectors [13, 19]. In this regard, the availability of transition of the workplace from office to home to prevent infection varied according to the working environment of each occupation [14]. In the case of occupations with no major restrictions on the working environment, it was relatively easy to work from home. However, for workers who have to work in a specific place and sales/service workers who have to interact with customers in person, it was impossible to work from home. In particular, service workers, whose jobs entailed facing their clients, were exposed to greater stress and fear of infection due to the changed working environment, in which adherence to the infection prevention guidelines was mandatory. Nevertheless, it was difficult to keep a distance from customers [17].

In this context, sales/service workers were more vulnerable to COVID-19 infection, although they faithfully adhered to personal preventive measures of social distancing. This is because of directly coming in contact with customers [15]. Accordingly, they were exposed to intense fear and stress, not only for their safety from infection but also due to the risk of spreading infection [16, 17]. Further, they were exposed to a higher risk of severe depression compared to other occupational groups [18]. A study on COVID-19-related depression among social workers—whose service involves face-to-face contact—found that the greater the extent to which their daily lives were disrupted, the more likely they were to experience depression, whereby COVID-related stress had a mediating effect [19]. These fragmentary research results, which may be considered a series of processes, highlight the importance of examining the paths of mental health outcomes according to occupation type in a disaster situation such as the COVID-19 pandemic.

Although some research has been conducted on COVID-19-related mental health, those studies have drawn only fragmentary predictive factors and mental health outcomes, with hardly any research dedicated to examining mental health outcomes by occupation [18]. Furthermore, existing studies on epidemic disasters have mostly focused on healthcare professionals among professionals dealing with clients directly [20–22], with little attention paid to the occupational risks of sales/service workers [23–25]. Therefore, this study examines how occupation type affects depression, a representative indicator of mental health, through a dual mediation of the changes in daily life and anxiety caused by COVID-19 as perceived by individual workers.

Thus, this study aims to shed light on the effects of occupation type (office worker/service worker) on depression through the mediation of the changes in daily life and anxiety as perceived by individual workers through their experiences of the COVID-19 pandemic. It aimed to understand the occupation-dependent differences in the process of social and psychological reactions of workers to special disasters (e.g., infectious disease) and explore clinical and institutional measures to promote mental health.

## Methods

### Research model

**Fig. 1:**
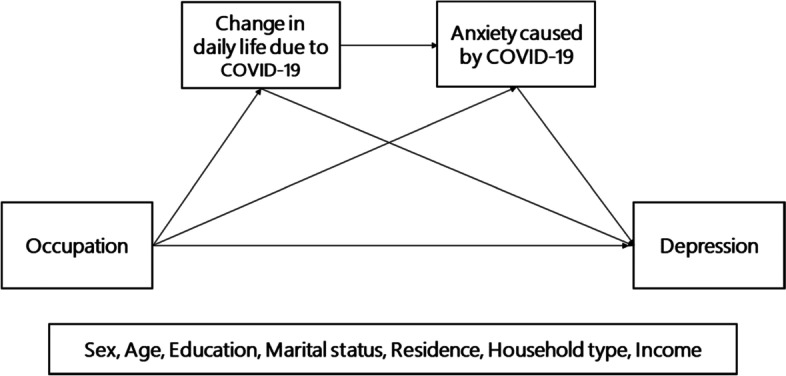
Research model

Figure 1 shows the research model proposed in this study to examine the effects of occupation type on depression through the dual mediation of the changes in daily life and anxiety as perceived by individual workers due to the COVID-19 pandemic.

### Research data and subjects

Raw data from the 2020 National Community Health Survey were obtained for the analysis of this study. The aforementioned survey is conducted every year by the Korea Centers for Disease Control and Prevention in cities-counties-districts administrative units across the country to assess local residents’ health status pursuant to Article 5 (Community Health Status Survey) of the Regional Public Health Act and Article 2 (Methods and Details of the Community Health Status Survey) of the Enforcement Decree of the Regional Public Health Act. The final selection of survey samples is subject to the approval of the Sampling Design Committee of the Korea Centers for Disease Control and Prevention [26]. For the analysis of this study, a total of 68,207 “office” and “service” workers were selected from the 2020 National Community Health Survey raw data on economically active adults (19 to 65 years).

### Variable measurements

#### Dependent variable: depression

Depression was analyzed using Patient Health Questionnaire-9 (PHQ-9), a self-reported depression screening instrument developed by Spitzer [27]. It has fewer items than other depression screening tools and has been found useful for screening and assessing depression symptoms. It comprises nine items (anhedonia, depressed mood, sleep problems, fatigue, appetite change, feelings of worthlessness & self-deprecation, concentration difficulties, psychomotor agitation, thoughts of suicide & self-harm). Each item is rated on a 4-point Likert scale (0 = not at all, 3 = nearly every day). The total score is a simple summation of all item scores, where a higher total score indicates a higher level of depression. The Cronbach’s α coefficient for the depression items of PHQ-9 was .777 in this study.

#### Independent variable: occupation type

For this study, we divided occupations into two types—“office work” and “service work”—considering the working environment and work characteristics based on the Korean standard classification of occupations. For analysis purposes, we coded the former into 1 and the latter into 0.

#### Mediator variables: changes in daily life due to COVID-19 and anxiety caused by COVID-19

The first mediator variable, COVID-19-related changes in daily life, was measured with an item rated on a scale of 100 (where 0 = a complete disruption of daily life routines and 100 = pre-COVID-19-level daily life), which was reverse-scored, with a higher score indicating a higher level of changes in daily life due to COVID-19.

The second mediator variable, anxiety caused by COVID-19, was measured with a 5-item self-reporting questionnaire, rating the levels of anxiety about COVID-19 infection and death of the self, blame and harm from others in case of infection, infection risk for family and those with vulnerable health conditions, and COVID-19-related economic damage for the self and family (losing a job or difficulty in finding a job). Each item rated on a 5-point Likert scale (5 = not at all, 1 = very much) was reverse-scored, with a higher score indicating a higher level of anxiety caused by COVID-19. The Cronbach’s α coefficient for anxiety caused by COVID-19 was .762 in this study.

#### Control variables

The control variables used for the study’s analysis are participants’ general characteristics—sex, age, education level, marital status, household type, monthly income, and residential region. For analysis purposes, they were classified and coded as follows: male (1), female (0); ≤Middle school (0), high school (1), ≥University (2); married (1), single (2), divorced/widowed/ separated (3); co-residential household (0), one-person household (1); metropolitan area (1), non-metropolitan area (0). Age and household monthly income were used as continuous variables, whereby the mean monthly income was transformed into a logarithmic form for analysis to address the problem of normality.

### Data analysis

Data analysis was performed using PROCESS Macro 4.0 for SPSS 25 to test the research model. First, descriptive statistics and correlation analysis were used to identify the typical characteristics of the survey participants. Second, PROCESS Macro (Model 6) was used to test the research hypothesis. Model 6 is useful for testing dual mediating effects of two serial parameters, dissimilar to simple-parameter or multi-parameters. Accordingly, research hypothesis testing was performed on the effect of the independent variable (occupation type) on the mediator variables 1 (changes in daily life due to COVID-19) and 2 (anxiety caused by COVID-9). Finally, regression analysis was performed in order of assessing the effects of the independent variable and mediator variables 1 and 2 on the dependent variable. Furthermore, bootstrapping was used to determine the degree of statistical significance of the mediating effects by drawing 5000 samples from the data to calculate a 95% confidence interval.

## Results

### Participants’ general characteristics

Table 1 presents the descriptive statistics of major sociodemographic characteristics of the survey participants used in this study—68,207 economically active adults aged 19–65 years. Analysis results for sex, education level, residential region, household type, marital status, and occupation type are as follows (in decreasing order of frequency): female (55.3%), male (44.7%); ≥ university (66.4%), high school (27.7%), ≤ middle school (5.9%); non-metropolitan areas (66.4%), metropolitan areas (41.6%); co-residential households (88.8%), one-person households (11.2%); married (64.1%), single (25.7%), divorced/widowed/separated (10.2%); service workers (61.5%), office workers (38.5%).

**Table 1:** Demographic characteristics of the participants

		(*N* = 68,207)
	N	%
Sex		
Female	37,745	55.3
Male	30,462	44.7
Education		
≤Middle school	4021	5.9
High school	18,905	27.7
≥University	45,281	66.4
Residence		
Metropolitan area	28,387	41.6
Non-metropolitan area	39,820	58.4
Household type		
Multiple	60,540	88.8
Single (One-Person)	7667	11.2
Marital status		
Married	43,714	64.1
Unmarried	17,518	25.7
Divorced/widowed/separated	6975	10.2
Occupations		
Office	26,230	38.5
Service	41,977	61.5
Continuous variable	M	SD
Age	43.8	11.802
(Log) Income	5.4	.729

### Correlation analysis and multicollinearity between the main variables

Table 2 presents the results of the correlation analysis between the main variables. The correlation coefficients between the main variables did not exceed the threshold of 0.8, with absolute values ranging from 001 to 0.637. Likewise, with the values of the variance inflation factors ranging from 1.040 to 2.203, well below the threshold of 10, there was no problem of multicollinearity.

**Table 2:** Correlation analysis

											(*N* = 68,207)	
	1	2	3	4	5	6	7	8	9	10	11	12
	1											
2.	.008*	1										
3.	.190**	−.406**	1									
4.	.062**	−.115**	.137**	1								
5.	.016**	−.049**	−.012**	−.011**	1							
6.	.009*	−.637**	.210**	.109**	.281**	1						
7.	−.117**	.221**	−.167**	−.037**	.282**	−.198**	1					
8.	.064**	−.027**	.174**	.068**	.017**	−.056**	−.162**	1				
9.	.136**	−.181**	.419**	.117**	−.007	.076**	−.119**	.159**	1			
10.	−.077**	−.029**	−.013**	.015**	−.006	−.013**	.001	−.032**	−.049**	1		
11.	−.159**	.100**	−.150**	−.084**	−.047**	−.107**	.043**	−.065**	−.106**	.176**	1	
12.	−.096**	−.107**	−.012**	.046**	.063**	.082**	.031**	−.054**	−.028**	.095**	.059**	1

1. Sex (ref. 1); 2. Age; 3. Education; 4. Residence(ref. 1); 5. Household type(ref. 1); 6. Marital status (ref. 2); 7. Marital status (ref. 3); 8.(log)income; 9. Occupations (ref. 1); 10. Changes in daily life due to COVID-19; 11. Anxiety caused by COVID-19; 12. Depression * *p* < .05, **p < .01

### Mediating and dual mediating effects of COVID-19-related changes in daily life changes and anxiety

For two mediator variables, as is the case with this research model, a three-step regression analysis is performed using Model 6 of PROCESS macro to analyze the simple and dual mediating effects. Accordingly, we analyzed the dual mediation of variations in daily life due to COVID-19 and anxiety caused by it in the mental health relationship between occupation and depression depending on the occupation type. The analysis results are presented in Table 3.

**Table 3:** Relationship between occupations and depression during the COVID-19 pandemic: dual mediating effects

								(*N* = 68,207)		
		Step 1: DV=Changes in daily life due to COVID-19			Step 2: DV = Anxiety caused by COVID-19			Step 3: DV=Depression		
		B	SE	t	B	SE	t	B	SE	t
Independent Variable	Occupations (ref. 1)	-2.199	0.190	−11.601***	−0.043	0.006	−6.905***	− 0.011	0.003	−4.051***
Mediator variables	Changes in daily life due to COVID-19				0.006	0.000	43.696***	0.001	0.000	18.942***
	Anxiety caused by COVID-19							0.017	0.002	10.737***
Control variables	Sex (ref. 1)	−3.198	0.172	−18.551***	− 0.180	0.006	−31.577***	− 0.044	0.002	−18.671***
	Age	−0.134	0.010	−13.405***	0.001	0.000	4.075***	−0.003	0.000	−22.488***
	Education	0.254	0.169	1.502	−0.098	0.006	−17.541***	−0.013	0.002	−5.742***
	Residence (ref. 1)	1.041	0.171	6.087***	−0.076	0.006	−13.526***	0.030	0.002	13.018***
	Household type (ref. 1)	1.172	0.299	3.928***	−0.057	0.010	−5.776***	0.054	0.004	13.209***
	Unmarried (ref. 1)	−1.369	0.309	−4.437***	−0.013	0.010	−1.271	0.018	0.004	4.387***
	Divorce/widowed/separated (ref. 1)	−3.446	0.270	−12.782***	− 0.102	0.009	−11.508***	− 0.002	0.004	−0.617
	Income	−0.930	0.119	−7.826***	−0.033	0.004	−8.514***	−0.017	0.002	−10.429***
Model fits		R2 = .112, F = 93.247***			R2 = .281, F = 569.948***			R2 = .175, F = 233.661***		

** *p* < .05, **p < .01, ***p < .001

Step 1 of Table 3 shows the results of multiple linear regression analysis regarding the effect of the independent variable on mediator variable 1 (changes in daily life due to COVID-19), which was performed as the first process step of data analysis in Model 6. The explanatory power of the model was 11.2%, which was statistically significant (F = 93.247, *p* < .001). In this model, the independent variable—occupation type—was found to have a negative effect on mediator variable 1—changes in daily life due to COVID-19 (B = − 2.199, *p* < .001). It meant that service workers had to face a higher level of perceived changes in daily life due to COVID-19 than office workers. Regarding the control variables, the following groups reported to perceive changes in daily life due to COVID-19 more acutely than the opposite groups: women (B = -3.198, *P* < .000), younger age groups (B = -.134, *P* < .000), metropolitan area (B = 1.041, *P*<. 000), one-person households (B = 1.172, *P* < .000), and lower- income (B = -.930, *P* < .000).

Step 2 of Table 3 shows the results of analyses regarding the effects of the independent variable (occupation type) and mediator variable 1 (changes in daily life due to COVID-19) on mediator variable 2 (anxiety caused by COVID-19), which were performed as the second process step of data analysis in Model 6. Step 2 analysis model was also statistically significant (F = 569.948, *P* < .001), whereby the independent variable had a negative effect on moderator variable 1 (B = -.043, *P* < .001), and moderator variable 1 had a positive effect on moderator variable 2 (B = .006, *P* < .001), both with statistical significance. This demonstrates that anxiety due to COVID-19 increased to a greater extent among service workers than among office workers and that the greater the extent of perceiving changes in daily life due to COVID-19, the greater the anxiety caused by COVID-19. Regarding the control variables, the following groups reported perceiving a higher increase in anxiety caused by COVID-19 compared to the opposite groups: women (B = -.180, *P* < .000), older age groups (B = .001, *P* < .000), a lower education level (B = -.098, *P* < .000), non-metropolitan areas (B = -.076, *P* < .000), co-residential households (B = -.057, *P* < .000), and lower- income (B = -.033, *P* < .000).

Finally, Step 3 of Table 3 shows the results of analyses regarding the effects of the independent variable and mediator variables 1 and 2 on the dependent variable, depression, which were performed as the third step of data analysis in Model 6. Similarly, Step 3 analysis model was statistically significant (F = 233.661, *P* < .001), whereby depression was verified to be significantly affected by occupation type (B = -.011, *P* < .001), changes in daily life due to COVID-19 (B = .001, *P* < .001), and anxiety due to COVID-19 (B = .017, *P* < .001). In other words, depression increased to a greater extent among service workers than among office workers. The greater the extent of perceiving changes in daily life due to COVID-19 and the higher the level of anxiety caused by COVID-19, the greater the increase in depression due to COVID-19. Furthermore, regarding the control variables, the following groups reported a greater increase in depression compared to the opposite groups: women (B = -.044, *P* < .000), younger age groups (B = -.003, *P* < .000), lower education level (B = -.013, *P* < .000), metropolitan area (B = .030, *P* < .000), one-person households (B = .054, *P* < .000), single (B = .018, *P* < .000), and lower- income (B = -.017, *P* < .000).

**Fig. 2:**
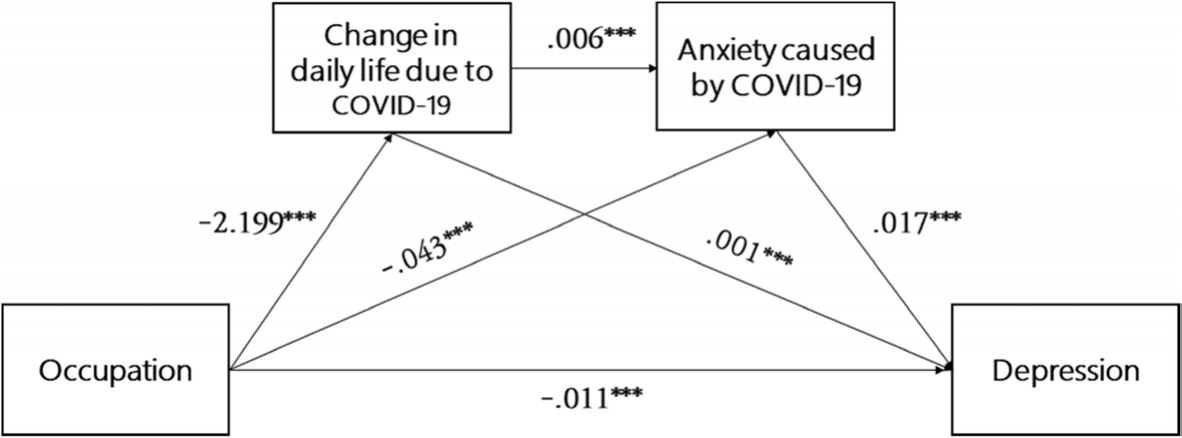
Result of Analysis Model

Figure 2 is a schematic summary of the analysis results for the dual mediating effects of COVID-19 related changes in daily life and anxiety on the relationship between occupation type and depression, as presented in Table 3.

Table 4 provides the results of statistical significance testing of mediating effects via the bootstrap method performed upon completion of the 3-step regression analysis performed in Model 6 of PROCESS macro. The results confirm the statistical significance of the overall mediating effect (.0032; Boot CI: −.0038 to −.0026), thus establishing the model fit of the dual mediation model evaluated in this study. Regarding the mediating effects of each path, the effect of occupation type on depression via mediator variable 1—changes in daily life due to COVID-19—was found to be statistically significant (Boot CI: −.0027 to-.0018), as was the effect via mediator variable 2—anxiety due to COVID-19 (Boot CI: −.0010 to −.0005). Similarly, the dual mediating effects, that occupation type has on depression through changes in daily life and anxiety due to COVID-19, were found to be statistically significant (−.0002; Boot CI: −.0003 to −.0002).

**Table 4:** Mediating effect

			(*N* = 68,207)	
	Effect	BootSE	Boot 95% CI	
			LLCI	ULCI
X → M1 → Y	−.0022	.0002	−.0027	−.0018
X → M2 → Y	−.0007	.0001	−.0010	−.0005
X → M1 → M2 → Y	−.0002	.0000	−.0003	−.0002
Total	−.0032	.0003	−.0038	−.0026

X: Occupations, M1: Changes in daily life due to COVID-19, M2: Anxiety caused by COVID-19, Y: Depression, *BootSE* Bootstrap Standard Error

## Discussion

The main study results and related discussion points can be summarized as follows.

First, changes in daily life due to COVID-19 were perceived more acutely among service workers than among office workers. This may be associated with the nature of their working environments—department stores and hypermarkets for the most part—where infection control measures should be adhered to more strictly and mandatorily due. This is because their work characteristics include services that require face-to-face interaction with the clients, unlike back-office working environments and work characteristics. Furthermore, more conservative restrictions on daily life can also be considered, given the elevated risk of workplace lockdown, others’ reproaches, and damage to family and acquaintances due to the nature of their work, which inevitably involves services that deal with face-to-face interaction with the clients.

Second, service workers perceived a higher level of anxiety caused by COVID-19 than office workers, with the level of anxiety increasing in proportion to that of their perceived changes in daily life due to COVID-19. These results support the research finding that service workers, who provide client-facing services, are vulnerable to COVID-19 infection, regardless of how carefully they comply with infection control measures at the individual level. This is because of the nature of their work, thus increasing the severity of their stress and anxiety from fear of infection [15–17].

Finally, it was found that service workers experienced changes in daily life due to COVID-19 pandemic to a greater extent than office workers, which had a positive effect on the level of anxiety caused by COVID-19, ultimately increasing depression. This result supports the research finding that people who experience difficulties in adapting to COVID-19-related societal changes as a whole are exposed to higher risks of stress, anxiety, and depression [19, 28–30]. This result also demonstrates that different occupations lead to different perceptions and experiences of COVID-19-related changes in daily life, given the context of various levels of individually perceived changes in daily life and their acceptance in special disaster situations such as the COVID-19 pandemic [13]. This allows the assumption that the working environment and work characteristics during the COVID-19 pandemic function as causes of perceived changes in daily life and anxiety as experienced by individual workers in this COVID-19 era. It also suggests that the actual objective changes and the subjective individual perceptions can be important predictors of negative mental health conditions such as anxiety and depression. In particular, the results of this study are the ones that determine the paths of psycho-emotional response to the effects of occupation type on depression. Thus, they fulfill the function of testing fragmentary evidence of occupation-dependent anxiety and mental health outcome and related state surveys [15, 16, 31–33] using a statistical model.

The clinical and policy implications of these results of this study from the health and welfare perspectives are as follows. First, various intervention programs should be developed to promote mental health customized to the type of occupation, with particular attention paid to working environment and work characteristics vulnerable to COVID-19 infection. For example, it would be worthwhile to prepare and provide various online/offline mental health intervention programs that consider the working environment and work characteristics of each occupation type in pandemic scenarios such as the COVID-19 as well as changes in daily life due to it. In particular, specialized programs, such as stress and anxiety coping techniques, may be considered for the purpose of relieving infection-related stress and anxiety stemming from additional workloads due to physical infection prevention guidelines and client-facing services. Second, in addition to psycho-emotional programs, physical environments need to be improved to better protect service workers’ physical and mental health. In this context, strategies to reduce client-facing time may be considered in times of disasters like the COVID-19 pandemic, such as allocating more break time or fewer customer service cases than in normal times. Moreover, companies, businesses, and employers will have to meticulously conduct and support public health and infection control measures to relieve their employees’ anxiety, shifting the emphasis from complying with infection control guidelines for customers/consumers. Furthermore, the central and regional governments will have to provide relevant public health education for employers and businesses that can help abate the stress and anxiety of workers in times of the pandemic.

This study is significant for exploring the psycho-emotional response process based on mental health outcomes considering the working environments affected by the COVID-19 pandemic, breaking away from the prior research approaches applied to COVID-19-related research on the general public [10]. Further, it is noteworthy as it compares the general mental health indicators before and after the COVID-19 pandemic. Additionally, it sheds light on the structural relationship regarding the effect of occupation type on depression through the dual mediation of changes in daily life due to COVID-19 and anxiety caused by COVID-19, specifically contrasting two occupational groups—client-facing sales/service work and telecommuting or less client-facing work.

Nevertheless, two aspects may be pointed out as limitations of this study. First, the COVID-19-related items used were not standardized scales or instruments. Moreover, a single-item scale and a limited number of variables were used for the working environment and work characteristics. Second, the study was limited to comparing two occupation groups, although there are many occupation groups other than office jobs and service jobs according to the occupation classification standard. To address these limitations, follow-up studies will have to comprehensively examine various occupational groups and reflect the indicators that can objectively measure the level of changes in daily life or other occupation-dependent working environment or work characteristics, thus drawing a wide range of discussions.

## Conclusions

This study examined the effects of occupation type on depression in this COVID-19 era through the dual mediation of COVID-19-related changes in daily life and anxiety. The study verified the dual mediating effects of those changes in daily life and anxiety in the relationship between occupation type and depression. This finding suggests that special disaster situations such as the COVID-19 pandemic may exert varying impacts on the perceived changes in daily life and level of anxiety and consequently on mental health, depending on the occupation type. Therefore, it is necessary to develop various customized mental health services and policies considering the employment environment and work characteristics of those vulnerable to COVID-19 infection.

## Data Availability

The dataset supporting the findings of this article is available upon reasonable request from the corresponding author.

## Abbreviation

PHQ-9: Patient Health Questionnaire-9
